# Supplementation of standard antibiotic therapy with oral probiotics for bacterial vaginosis and aerobic vaginitis: a randomised, double-blind, placebo-controlled trial

**DOI:** 10.1186/s12905-015-0246-6

**Published:** 2015-12-03

**Authors:** Piotr B. Heczko, Anna Tomusiak, Paweł Adamski, Artur J. Jakimiuk, Grzegorz Stefański, Aleksandra Mikołajczyk-Cichońska, Magdalena Suda-Szczurek, Magdalena Strus

**Affiliations:** Chair of Microbiology, Jagiellonian University Medical College, Krakow, Poland; Institute of Nature Conservation, Polish Academy of Sciences, Krakow, Poland; Clinical Department of Obstetrics and Gynecology, Central Clinical Hospital of Ministry of Interior and Administration, Warsaw, Poland; Department of Reproductive Health Research Institute of Mother and Child, Warsaw, Poland; IBSS BIOMED S.A, Krakow, Poland

**Keywords:** Probiotics, *Lactobacillus*, Bacterial vaginosis, Aerobic vaginitis

## Abstract

**Background:**

This multicentre, randomised, double-blind, placebo-controlled trial was performed to determine whether the use of oral probiotic preparation (prOVag®) containing three *Lactobacillus* strains together with standard metronidazole treatment and also targeted antibiotic treatment (following the failure of metronidazole therapy) could reduce the recurrence rates of bacterial vaginosis (BV) and aerobic vaginitis (AV).

**Methods:**

Patients at private gynaecological clinics in Poland with histories of recurrent BV/AV and current symptoms were randomly allocated to receive metronidazole and probiotic or placebo, and assessed monthly on visits II and III-V. The total number of study visits was 5–6 (I, II, II bis – if applicable, III, IV, V). One probiotic or placebo capsule was administered with metronidazole/targeted antibiotic twice daily for 10 days; during follow up, patients took one capsule daily for 10 days perimenstrually. Clinical examination and vaginal swabbing were performed at each visit. Primary outcomes were clinical or microbiological BV/AV recurrence and probiotic safety. Secondary outcomes were vaginal pH, Nugent score, and *Lactobacillus* counts in the vaginal microbiota. Safety analysis was performed in 578 (probiotic, *n* = 285; placebo, *n* = 293) 18–50-year-old women who were randomised.

**Results:**

BV/AV was confirmed microbiologically in 241 (probiotic, *n* = 118; placebo, *n* = 123) participants, who continued the trial. Data from 154 (probiotic, *n* = 73; placebo, *n* = 81) participants who completed the study were analysed to determine the efficacy of prOVag. Additional analyses included 37 (probiotic, *n* = 22; placebo, *n* = 15) participants who received targeted antibiotics and probiotics or placebo. prOVag lengthened the time to clinical relapse of BV/AV symptoms up to 51 % (*p* < 0.05) compared with placebo; AV relapse was delayed by up to 76 % (*p* < 0.05). Probiotic use also reduced and maintained low vaginal pH and Nugent score, and increased vaginal *Lactobacillus* counts following standard treatment.

**Conclusion:**

This study demonstrated that oral probiotics lengthened remission in patients with recurrent BV/AV and improved clinical and microbiological parameters.

**Trial registration:**

NCT01993524; 20 November 2013.

**Electronic supplementary material:**

The online version of this article (doi:10.1186/s12905-015-0246-6) contains supplementary material, which is available to authorized users.

## Background

Bacterial vaginosis (BV) is the most common vaginal infection in women of reproductive age [1], caused by overgrowth of anaerobic bacteria with reductions in *Lactobacillus* populations in the vagina [2]. Aerobic vaginitis (AV), referred to as ‘intermediate flora’, is related to the suppression of lactobacilli by various aerobic bacteria [3–5]. AV is often found in patients treated unsuccessfully for BV with antibiotics [6]. The standard therapy for BV is oral metronidazole, vaginal clindamycin cream, or metronidazole gel [7], whereas targeted therapy according to an individual’s antibiotic resistance is recommended for AV [6]. Treatment success rates of about 50 % have been reported for BV and AV, and recurrence rates can exceed 50 % within 6–12 months after treatment cessation [8, 9]. Most proposed non-antibiotic therapies for BV are vaginal or oral probiotics, which aim to restore the normal vaginal microbiota [2]. Several trials evaluated the use of combined metronidazole and/or clindamycin therapy and vaginal probiotics to prevent BV recurrence. In a study by Larsson et al. [10], BV was treated with clindamycin cream and, subsequently, a vaginal probiotic containing *L.gasseri* and *L.rhamnosus* or placebo. Use of the probiotic contributed to prevention of relapse (hazard ratio = 0.73). Marcone et al. [11] treated 24 women with BV initially with oral metronidazole, and then with vaginal doses of *L.rhamnosus* for 6 months. Efficacy was evaluated solely using microbiological data. Abnormal vaginal microflora was found in 31 % subjects after 12 months compared with 9 % directly after metronidazole treatment in control group (significant increase in the number of women with abnormal flora over time). The same follow-up study showed no statistically significant difference in vaginal microflora in women from probiotic group (9 % during 12 months follow-up vs. 4 % directly after metronidazole). Ya et al. [12] conducted a study, in which 58/120 women with histories of recurrent BV were given vaginally *L.rhamnosus, L.acidophilus*, and *S.thermophilus* daily for 2 months, while 62 women received placebo. After 11 months, study subjects were interviewed by telephone about BV symptoms; 15.8 % of women who used the probiotic and 45 % of control subjects reported symptoms of BV. Delia et al. [13] published a report on a 3-month administration of a vaginal probiotic containing *L.acidophilus* or the same probiotic plus oral *L.paracasei* to women with suspected BV or BV confirmed by Amsel’s criteria. The authors concluded that the vaginal probiotic reduced pH and improved other parameters, and that the adjuvant oral probiotic sustained this effect for 6 months. Results of a recent double-blind, randomised, placebo-controlled clinical trial [14] on treatment of BV by oral application of two probiotic bacteria described earlier by Reid [15] show restoration of vaginal microbiota balance within 44 days in 61.5 % of patients receiving the probiotic, compared with nearly 27 % of the placebo group. Unfortunately, the diagnosis of vaginal microbiota balance was based only on a presumptive detection of lactobacilli based on four phenotypic criteria, one of which was misleading because a considerable proportion of *Lactobacillus* species produce catalase [16]. As stated by Hay, advances in our understanding of the pathogenesis of bacterial vaginosis allow the opportunity to improve treatments to prevent recurrence, which may require a combination of modalities [17]. As emphasised in a recent Cochrane review [18], large, randomised, placebo-controlled trials with standardised outcomes are needed to confirm the efficacy of probiotic/antibiotic treatment for BV. The efficacy of such combination treatment for AV has not been examined.

Thus, the aim of this study was to investigate whether the use of oral probiotic preparation prOVag together with standard treatment for BV or AV could reduce the recurrence rates, as per clinical and microbiological criteria, in comparison with standard treatment alone. prOVag is a preparation containing strains of three lactic acid bacteria: *L.fermentum* 57A, *L.plantarum* 57B, and *L.gasseri* 57C. All strains were isolated from a vaginal swab taken from the same healthy woman aged 26 years not using antibiotics for the last 3 months. The strains possessed high co-aggregating abilities and naturally occurred as a triad strains complex. Their species designation was confirmed by PCR for 16S RNA using species-specific primers and they were identified using both PFGE and MLST methods to distinguish them from other strains in materials taken during clinical study. They have been deposited in the internationally recognized collection and covered with a patent. They had been selected for commercial use on the basis of high adherence ability to human Caco-2 enterocytes and A431 vaginal cell lines, ability of selected vaginal pathogens adhesion reduction already adhered to these lines and a broad antagonistic properties exerted against *G.vaginalis, S.agalactiae*, *P.bivia*, *S.aureus*, *E.faecalis*, *C.difficile* and uropathogenic *E.coli. L.gasseri* 57C produces hydrogen peroxide. All of them are resistant to metronidazole and ciprofloxacin. *L.fermentum* 57A and *L.plantarum* 57B are vancomycin-resistant while *L.gasseri* 57C is sensitive to vancomycin. The strains carry no extrachromosomal DNA elements able to transmit resistance to antibiotics, and they are resistant to gastric juice with pepsin at pH 2.5, to bile salts and to spermicides like as nonoxynol-9.

## Methods

This multicentre, randomised, double-blind, placebo-controlled, parallel–group study was conducted between March 2009 and February 2012. Nine private outpatient gynaecological clinics in Poland recruited the patients. The trial received ethical approval from the independent ethics committee of the Silesia Medical Chamber, relevant to the study coordinator (no. 4/2009, 2 February 2009). The study protocol number is PB-DM/SBK–prOVag2–01/08. All participants provided written informed consent.

The sample size was calculated with the assumption that the product would be ≥50 % more efficient than placebo [19]. Previous studies have documented recurrence rates of ~40 % within 3 months following the end of treatment [20, 21]; thus, this recurrence rate was expected in subjects on placebo and the probiotic product was expected to reduce the recurrence rate to 20 %. The sample size was calculated with the pwr procedure in the R software package (version 2.15.0; R Development Core Team, 2012). Under these assumptions, the minimum sample size was determined to be 148 patients (74 per group) who successfully complete the clinical study. In recognition of several factors that may reduce the sample size, the screening group was increased to about 600 participants (probiotic group, *n* = 285; placebo group, *n* = 293).

Eligible participants were 18–50-year-old European descent women who menstruated regularly and had histories of recurrent BV. Table 1 lists the inclusion and exclusion criteria. Participants were asked not to use any new intimate hygienic product or vaginal douche during the trial. Participants recorded safety information in ‘patient’s diaries’, which an investigator analysed and documented at each follow-up visit.

**Table 1:** Inclusion and exclusion criteria applied in the study

Inclusion	Exclusion
At least three of the following BV symptoms, according to Amsel’s criteria [13]: homogenous vaginal discharge, vaginal pH > 4.5, positive amine test findings, and presence of clue cells in a wet smear observed under microscopy.	Women were ineligible if they were pregnant or breastfeeding; had known hypersensitivity to the investigated product or to metronidazole or other antibiotics, *Candida* vaginitis, bleeding from the genital tract of unknown aetiology, any pathology of the reproductive organs, congenital or acquired immunodeficiency, diabetes, mental illness, or neoplastic disease; used mechanical contraceptives, such as diaphragms, intrauterine contraceptive inserts, or hormonal vaginal rings; had used oral hormonal preparations or vaginal oestrogens in the reproductive period; were using another oral and/or vaginal probiotic at the time of assessment for inclusion in the study; had participated in another clinical study within the previous 30 days; were receiving antibiotic therapy for another reason; or were scheduled for surgery or hospitalisation.

A clinician examined all participants, recorded medical histories and clinical signs confirming a vaginal bacterial condition (BV/AV), and collected high vaginal swabs for further microbiological analysis performed in the central laboratory. Clinical examination and vaginal swabbing were performed at each visit as well as vaginal pH value that was measured by the investigator with pH indicator (Merck, range: 4.0-7.0). One vaginal swab from each participant was used to estimate Nugent scores [22]. The second vaginal swab was transferred from transport medium (Amies, Deltalab, Barcelona, Spain) to 1 ml Man, Rogosa, and Sharpes (MRS) broth (Difco Laboratories Inc., Detroit, MI,USA) and agitated for 1 min. Serial decimal dilutions in the same broth were then made, and 100-μl aliquots were plated on standard media for cultivation of aerobic and anaerobic bacteria and Candida yeasts. Bacteria were cultivated at 37 °C in aerobic or aerobic atmosphere for 24 or 48 hours. *G.vaginalis* isolates were identified on the basis of haemolysis on human blood agar, Gram-stained morphology showing typical Gram-negative rods, and negative catalase production. *Streptococcus agalactiae*, *S.bovis*, *Escherichia coli*, *Enterococcus* spp., and strict anaerobes such as *Prevotella bivia* were identified using standard phenotypic methods (API STREP, API STAPH, API 20E, and API 20A; bioMerieux). Only those microbial species or genera, found in cultures from vaginal swabs in numbers exceeding 10^5^ c.f.u. per swab, were regarded as related to vaginal infection [23].

*G. vaginalis* strains were tested for resistance to metronidazole and clindamycin using E-tests (bioMerieux). The resistance of common aerobic vaginal pathogens was tested using EUCAST disk diffusion method [24] for the following: azithromycin, doxycycline, amoxicillin, amoxicillin with clavulanic acid.

All eligible women were given at I visit a standard treatment (500 mg oral metronidazole twice daily for 7 days) and were randomly assigned to one of two study arms (1:1) (using block randomisation with a block size of 12 and equal group ratios) to receive probiotic or placebo twice daily for 10 days. At II visit (about 14 days after probiotic or placebo treatment cessation), participants were checked for symptoms and signs of vaginal infection and their vaginal swabs were analyzed for the presence of pathogens. If both, symptoms and *G.vaginalis*, were still present, metronidazole-resistant *G.vaginalis* BV infection was confirmed microbiologically and the patients were given oral clindamycin. When the infection signs and aerobic bacteria were found, AV diagnosis was made and an antibiotic indicated by individual susceptibility testing was used. Antibiotic was taken together with probiotic or placebo twice daily for 10 days.

Participants without signs of the infection during II visit were given only the probiotic or placebo once daily for 10 days in the peri-menstrual period (starting from 18 to 22 day of menstrual cycle) for the next 3 months. Participants with targeted antibiotic ordered on visit II were to come to the visit II bis, and if secondary treatment was successful they were to proceed for the next 3 months in the same manner as described above. Visits III-V occurred for all patients within ~7 days after completion of each menstrual period.

The probiotic used was a commercially available preparation (prOVag®) provided by the study sponsor (IBSS BIOMED S.A.,Krakow,Poland) containing a mixture of three viable strains: *L.gasseri* 57C, *L.fermentum* 57A, and *L.plantarum* 57B in a total number of ≥10^8^ c.f.u. Placebo looked identical but contained excipients only. Metronidazole and targeted antibiotics were licensed products provided by the study sponsor.

Recurrence of BV/AV at visits III-V was the study endpoint which means that patients diagnosed with a clinical relapse of BV/AV at visits III-V ended their participation in the study on the day of the visit when the diagnosis was made. However, if the reoccurrence of BV/AV was diagnosed at visit II (as a consequence of not efficient treatment), the patient stayed in the study. Nonetheless BV/AV diagnosis at visit II bis meant withdrawal of the study for the participant. The numbers and types of adverse events and serious adverse events were also assessed. The reduced probability of recurrent bacterial vaginal infection, confirmed by clinical symptoms and/or microbiological results, was the parameter used to assess primary efficacy. Secondary parameters were normalisation and maintenance of vaginal pH, Nugent score, and presence of >10^5^ c.f.u. of *Lactobacillus* in the vaginal flora per swab.

Data obtained at visits III-V were compared with those obtained at visit II. The time to recurrence was measured from the visit on which a participant showed no BV/AV symptom: visit II for participants on metronidazole treatment and the visit IIbis for participants treated with metronidazole and targeted antibiotics.

Statistical analysis were performed with JMP® software (version 7.0.1, SAS Institute Inc., Cory, NC, USA) and the R2.7.2 software package (R Foundation for Statistical Computing). Quantitative variables were compared between groups using Student’s *t*-test, or non-parametric (Mann–Whitney or Wilcoxon) tests in the case of non-normal distribution. For comparisons of more than two groups, analysis of variance with a *post-hoc* Tukey’s test was used. The non-parametric equivalents of these tests (Kruskal–Wallis and Steel–Dwass tests) were used to analyse variables that did not comply with the assumptions of analysis of variance. Data obtained at multiple visits were compared using parametric (Student’s *t*-test for matched pairs) or non-parametric (Wilcoxon signed rank) matched-pairs tests. Categorical variables were analysed using frequency (chi-squared or likelihood ratio) tests. The significance level for statistical analysis was set at *p* < 0.05.

## Results

The safety analysis included 578 participants in the intent-to-treat population, 241 (prOVag group, *n* = 118; placebo group, *n* = 123) of whom complied with the recommended regimen and completed treatment (i.e.used ≥20 capsules). No participant was excluded from the study due to an adverse event, and no serious adverse event related to the use of the probiotic product occurred. In total, there were 431 adverse events in 160 participants, which accounts for 28 % of the population for which the safety analysis was carried out (ITT); prOVag was used by 77 of these participants. There were no significant differences between the total numbers of adverse events reported in either of the study groups. Over half of all reported adverse events were considered to be unrelated to the use of the investigational product. Most of the reported adverse events concerned disorders of the gastrointestinal system, reproductive system and breasts. Main disorders of the gastrointestinal system included events possibly related to the use of metronidazole. The analysis of the time of their occurrence demonstrated that they were mainly reported at visit II, i.e. directly after the period when the participant used metronidazole. The statistical analysis of data did not demonstrate a significant correlation between using the investigational product by the participants and the occurrence of adverse events.

**Fig. 1:**
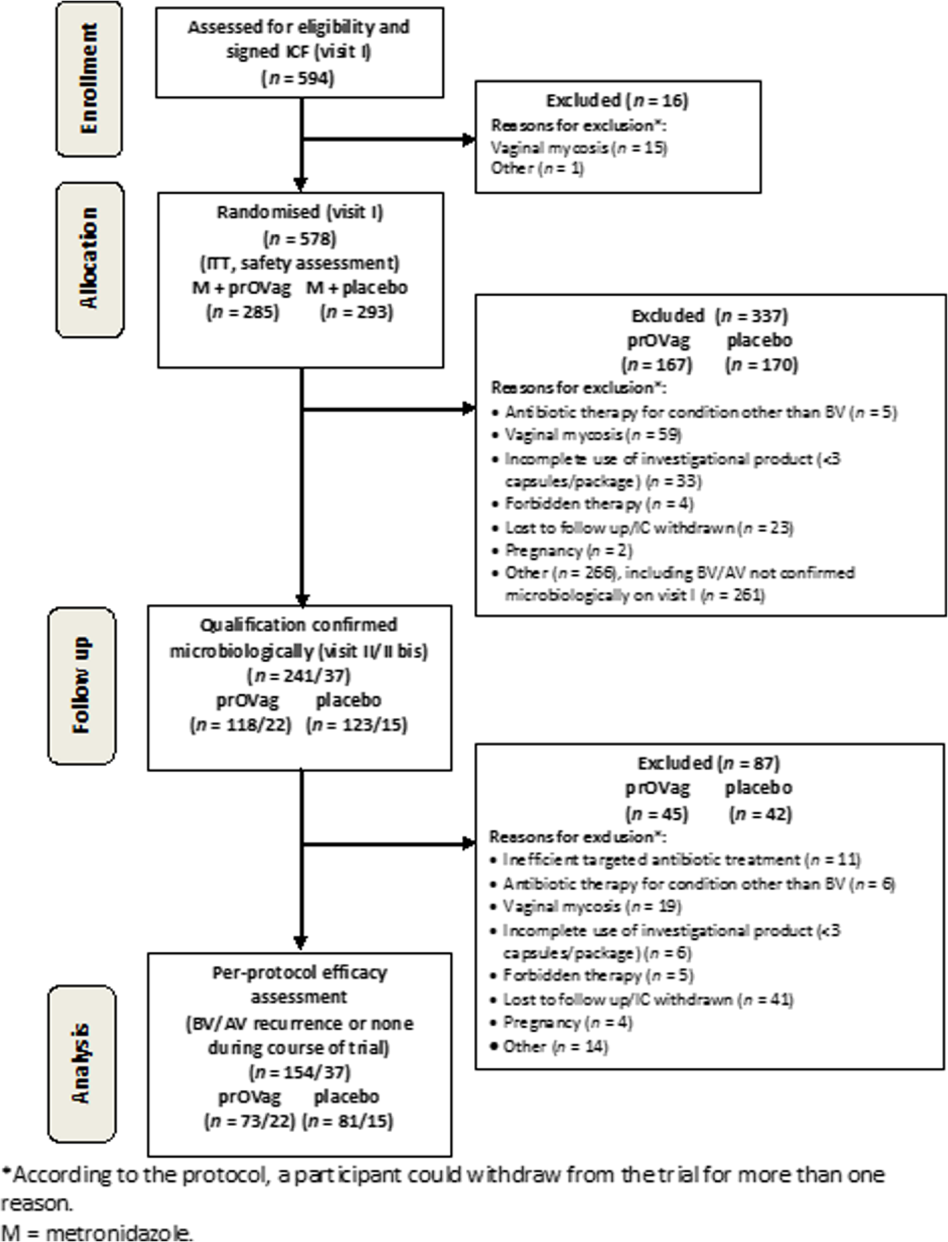
Participant flow

Table 2 presents the participants’ baseline characteristics. No significant difference in these characteristics between groups was found. Safety analysis was performed on the group of 578 participants who used at least one capsule of tested product (intent to treat-ITT) and the efficacy analysis was performed on the population who completed the full cycle of visits (*n* = 154, per protocol-PP). Figure 1 shows a diagram of participant flow.

**Table 2:** Baseline characteristics^a^ of patients in the intent-to-treat (ITT; *n* = 578) and per-protocol (PP; *n* = 154) populations

Characteristic	Placebo	Probiotic	*p*
Hormonal treatment ITT	15	20	0.3388
Hormonal treatment PP	3	5	0.3798
Contraception ITT	89	91	0.6866
Contraception PP	26	20	0.5244
Contraceptive tablets ITT	66	71	0.4999
Contraceptive tablets PP	16	13	0.8095
Transdermal contraception ITT	10	6	0.3380
Transdermal contraception PP	2	0	0.2047
Contraception condom ITT	10	8	0.6750
Contraception condom PP	5	4	0.9480
Contraception other ITT	4	6	0.4951
Contraception other PP	3	3	0.9420
Sexually active ITT	270	266	0.5838
Sexually active PP	76	70	0.5646
More than one sexual partner ITT	1	1	0.9844
More than one sexual partner PP	0	0	
Smoking ITT	46	32	0.1120
Smoking PP	14	12	0.8888
Vaginal douching ITT	2	3	0.6310
Vaginal douching PP	1	1	0.9411
Complaints at first visit (PP)			
Vaginal itching	7	6	0.9249
Vaginal burning	14	14	0.7301
Vaginal pain	1	1	0.9410
Vaginal discharge	79	70	0.5663
Pelvic pain	2	1	0.6221
Conditions reported by investigators at first visit (PP)			
Vaginal erythema	0	3	0.0354
Palpation tenderness	1	1	0.9624
Homogenous vaginal discharge	78	71	0.9260
Clue cell presence	79	71	0.9160
Positive amine test	78	72	0.3632
BV (Amsel’s criteria [13])	80	72	0.2559
Vulvovaginal candidiasis	0	1	0.2206
Microbial species cultured from vaginal swabs collected at first visit ^b^(PP)			
* Gardnerella vaginalis*	46	52	0.0628
* Streptococcus agalactiae*	17	15	0.9465
* Escherichia coli*	5	3	0.5646
* Enterococcus* spp.	0	1	0.2206
* Candida albicans*	2	0	0.1076
* Lactobacillus* spp*.*	48	44	0.8980

^a^Analyses were not carried for age, sex, or race, as only Caucasian women aged 18–50 years were included

^b^Only populations exceeding 10^5^ organisms per swab were counted

**Fig. 2:**
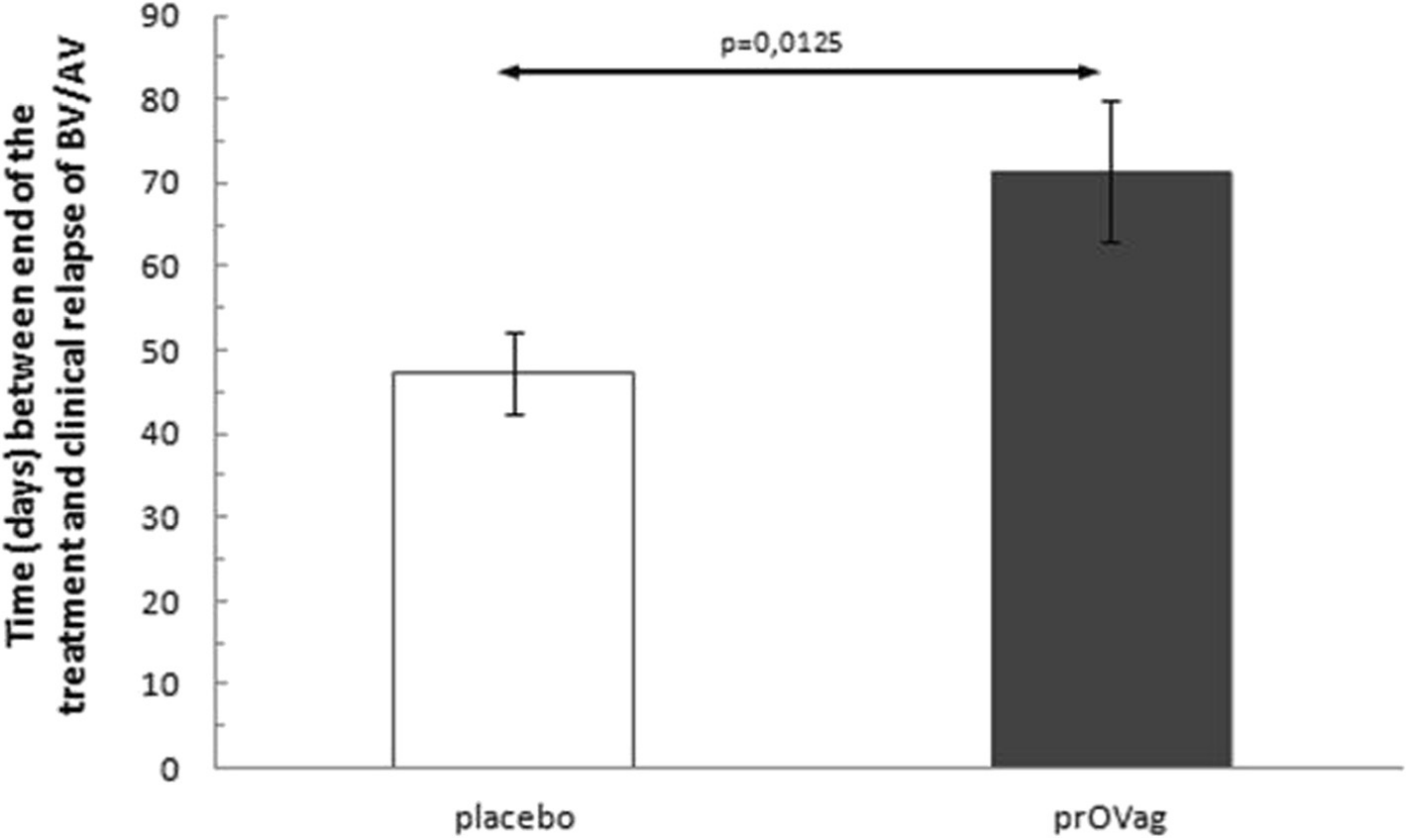
Interval between end of treatment and appearance of clinical relapse in patients on standard therapy taking prOVag or placebo

Relapse rates based on clinical symptoms didn’t show any significant differences between placebo and prOVag groups. However, the average times to relapse were 47.3 [standard deviation (SD) = 26.98] days in the placebo group and 71.4 (SD = 37.51) days in the prOVag group. This interval, which was up to 51 % longer in the prOVag group, differed significantly between placebo-treated and control patients (*t* = 2.606, *p* = 0.0125; Fig. 2).

**Fig. 3:**
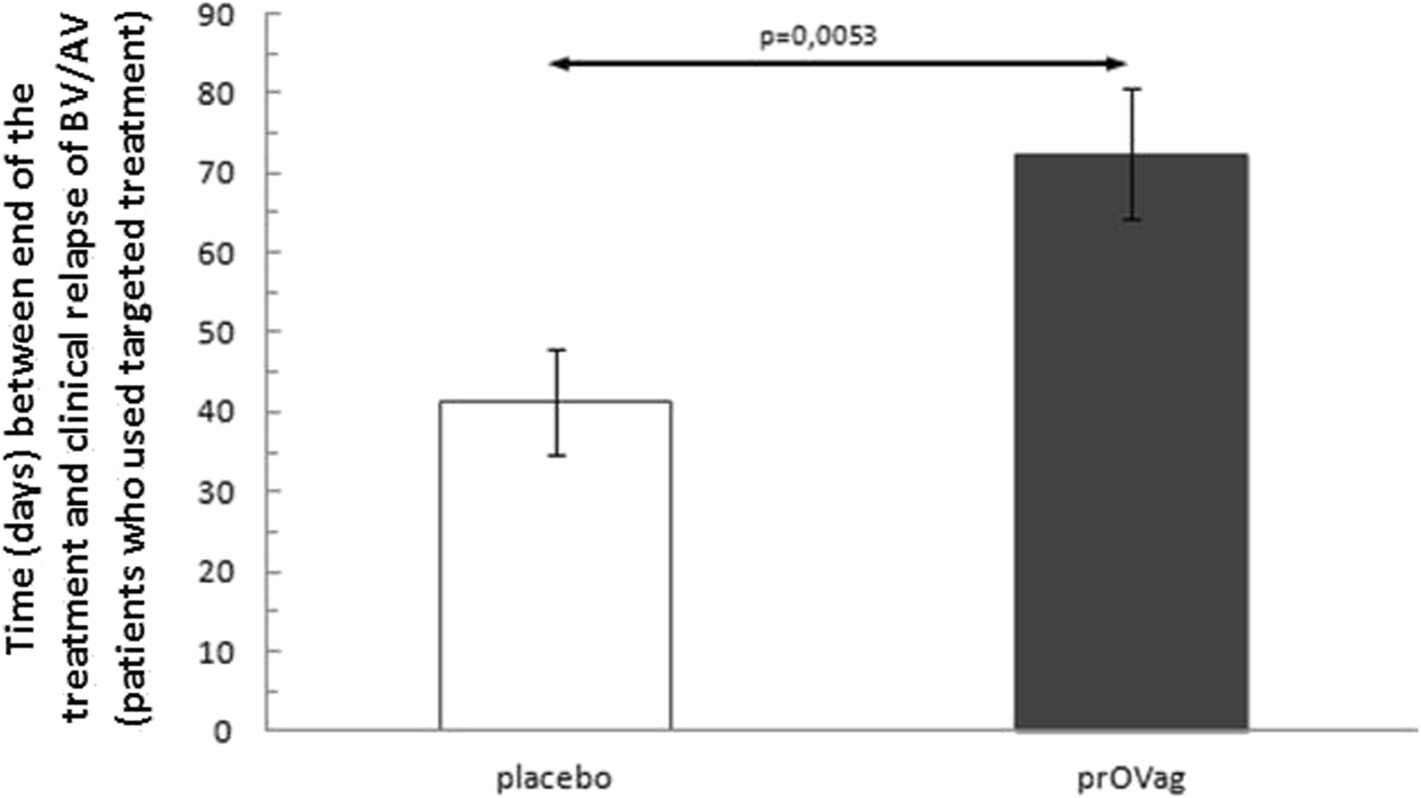
Interval between end of treatment and clinical relapse in patients receiving targeted antibiotics and prOVag or placebo

Fifty one participants showed BV/AV clinical relapse, 37 of them (prOVag group, *n* = 22; placebo group, *n* = 15) received secondary targeted treatment since either aerobic bacteria or metronidazole-resistant *G.vaginalis* were isolated. The intervals between the end of treatment and relapse in these patients were similar to the entire group: the time to relapse was up to 76 % longer in the prOVag group (72.4 ± 37.87 days) than in the placebo group (41.2 ± 24.86 days; *t* = 2.983324, *p* = 0.0053; Fig. 3).

Analysis of bacteriological data collected at visit I demonstrated that clinical symptoms were related most frequently to the presence of *G.vaginalis* (*n* = 98 patients), followed by *S.agalactiae* (*n* = 32), *E.coli* (*n* = 8), *Enterococcus* spp*.* (*n* = 1), and other bacteria (e.g. *P.bivia*, *S.bovis*)*.*

The relapse rate diagnosed on the basis of microbiological criteria (presence of >10^5^ c.f.u./swab of any bacterial species potentially related to BV/AV at visits III-V), occurred in 71 patients [prOVag group, *n* = 33 (45.2 %); placebo group, *n* = 38 (47.0 %)]. This difference approached statistical significance (*χ*^2^ = 4.883, *p* = 0.0870).

**Fig. 4:**
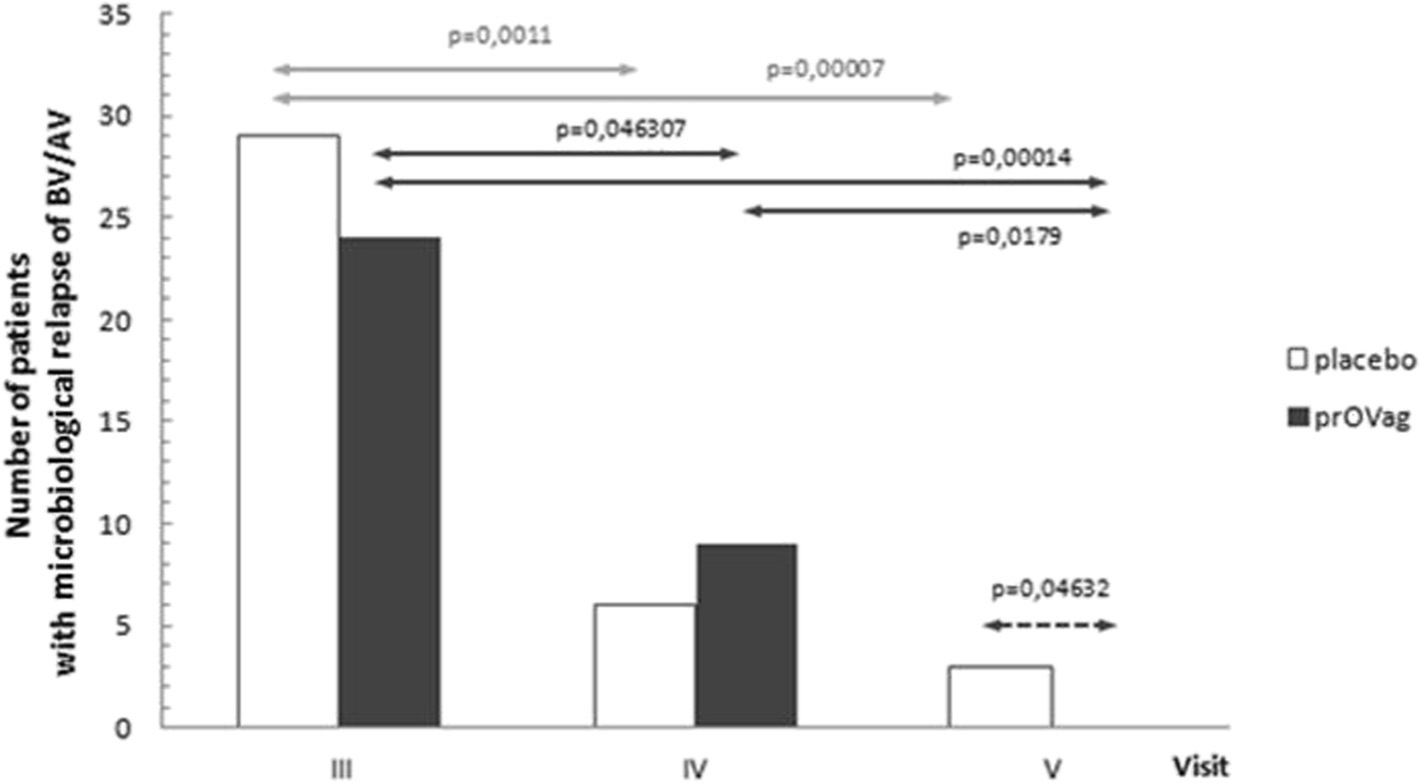
Number of patients with microbiological relapse detected at consecutive visits

In both study groups, the incidence of abnormal vaginal microbiota (*G.vaginalis* or aerobic pathogens) decreased at subsequent visits. The incidence of microbiologically confirmed BV/AV decreased significantly and progressively from visit III to visit V in the prOVag group, whereas significant reductions were observed only through visit IV in the placebo group. Thus, significantly fewer patients in the prOVag group had microbiologically confirmed BV/AV at visit V, compared with the placebo group (*G*^2^ = 3.9706, *p* = 0.04632; Fig. 4).

**Fig. 5:**
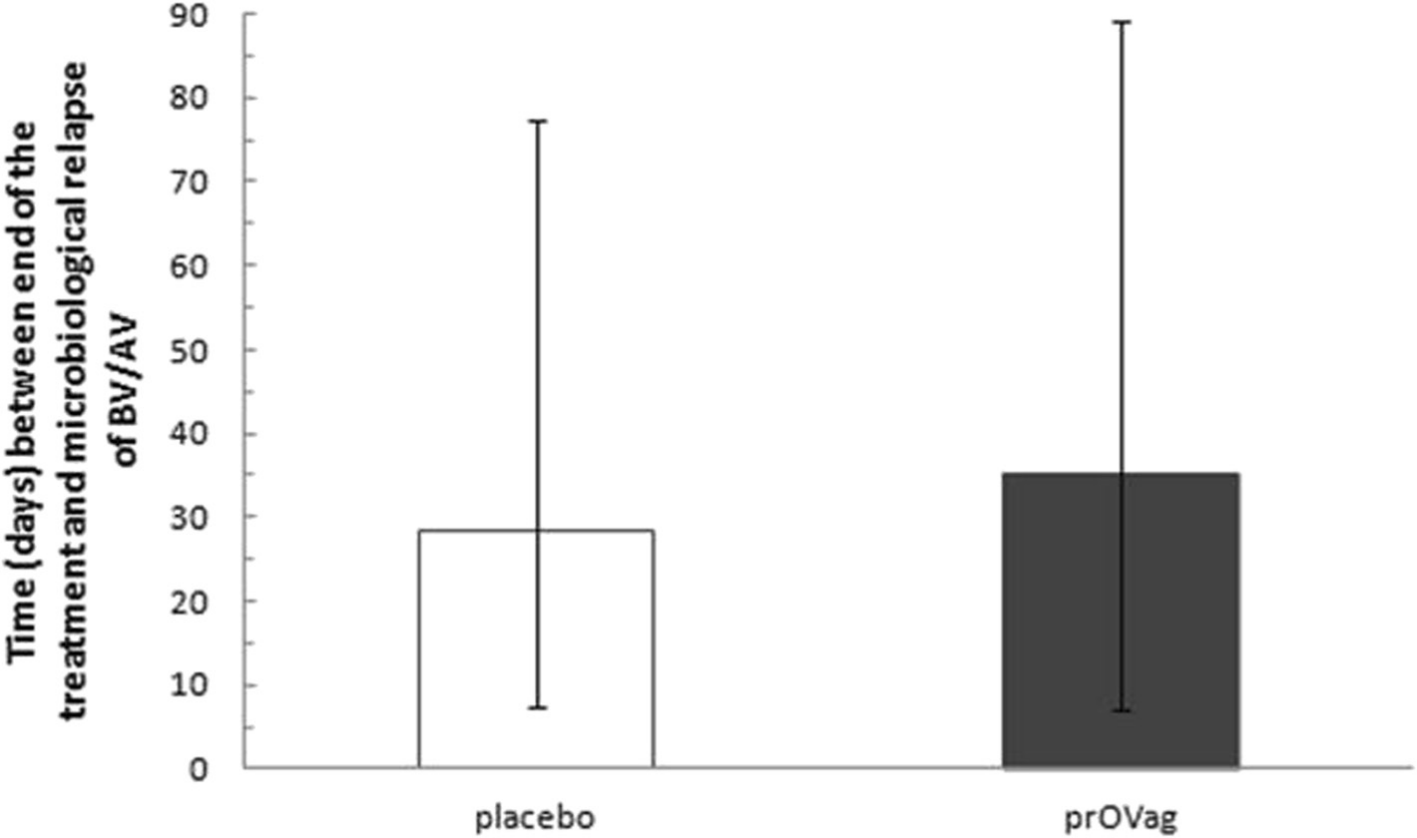
Interval between end of treatment and microbiological relapse in patients receiving antibiotics and taking prOVag or placebo

Because the interval between the end of treatment and relapse of microbiologically confirmed BV/AV was not normally distributed (*W* = 0.868216, *p* < 0.00001), the non-parametric Wilcoxon test was used. The interval in which no abnormal microbial flora was detected in the vagina was longer in the prOVag group (35.0 days; GK = 54, DK = 28) than in the placebo group (28.5 days; GK = 49, DK = 21), but this difference was not significant (*z* = 0.60352, *p* = 0.5426; Fig. 5).

Mean vaginal pH values decreased between all visits in the prOVag group, whereas slight increases in pH values were observed between visits III and V, and IV and V visits in the placebo group. Between visits IV and V, a mean increase of 0.18 units in the placebo group, and a mean decrease of 0.03 units in the prOVag group were observed; differences in pH changes between groups were significant (Table 3).

**Table 3:** Mean changes in vaginal pH between visits

	I vs III	I vs IV	I vs V	III vs IV	III vs V	IV vs V	Vis. I	Vis. III	Vis. IV	Vis. V
							Mean pH values SD			
placebo	Mean = −0.43	Mean = −0.61	Mean = −0.59	Mean = −0.08	Mean = 0.03	Mean = 0.18	5.24	4.77	4.65	4.63
	SD = 0.4626	SD = 0.808	SD = 0.499	SD = 0.628	SD = 0.562	SD = 0.725	0.303	0.459	0.434	0.377
prOVag	Mean = −0.52	Mean = −0.51	Mean = −0.59	Mean = −0.005	Mean = −0.06	Mean = −0.03	5.25	4.69	4.73	4.65
	SD = 0.806	SD = 0.701	SD = 0.567	SD = 0.564	SD = 0.557	SD = 0.386	0.308	0.651	0.480	0.444
	t = 0.660; *p* = 0.5112	t = 0.638; *p* = 0.5252	t = 0.031; *p* = 0.9753	t = 0.824; *p* = 0.4113	t = 0.829; *p* = 0.4091	t = −2.038; *p* = 0.0447	-	-	-	-

*SD* standard deviation

Changes in Nugent scores were not normally distributed (*W* = 0.9047; *p* < 0.0001) and were analysed using Kruskal–Wallis test. The scores differed significantly between groups throughout the study period (*H*_placebo_ = 115.874, *p*_placebo_ < 0.0001; *H*_provag_ = 87.392, *p*_provag_ < 0.0001). However, *post-hoc* Steel tests demonstrated that Nugent scores obtained at visit I did not differ significantly from those obtained at visits III–V in either group. As in the analysis of pH, matched-pair tests were used to eliminate the effects of individual differences between patients. In both groups, the Nugent scores tended to decrease over time; only differences between visits I and III, and III and IV were significant in the placebo, whereas the differences between visits I and III, I and IV, IV and V were significant in the prOVag group (Table 4). After the exclusion of patients in whom no change was observed, the probability of a reduction in the Nugent score between visits IV and V was significantly higher in the prOVag than in the placebo group (*χ*^2^ = 3.937, *p* = 0.0472).

**Table 4:** Changes in Nugent scores at subsequent visits

	Visit III	Visit IV	Visit V	Mean	SD
Placebo					
Visit I	H = 702.0 ↓	H = 770.0 ↓	H = 780.0 ↓	6.3	1.68
	*p* <0.0001	*p* < 0.0001	*p* < 0.0001		
Visit III	—	H = 150.0 ↓	H = 200.0 ↓	3.0	2.54
		p = 0.0037	p =0.0083		
Visit IV	—	—	H = 23.0 ↓	2.1	2.01
			*p* = 0.716		
Visit V	—	—	—	1.7	2.18
prOVag					
Visit I	H = 632.0 ↓	H = 642.5 ↓	H = 678.0 ↓	6.2	1.92
	*p* < 0.0001	*p* < 0.0001	*p* < 0.0001		
Visit III	—	H = 33.5 ↓	H = 106.00 ↓	2.9	2.58
		*p* = 0.4484	*p* = 0.0726		
Visit IV	—	—	H = 131.0 ↓	2.4	2.50
			*p* = 0.0201		
Visit V	—	—	—	1.8	2.52

**Fig. 6:**
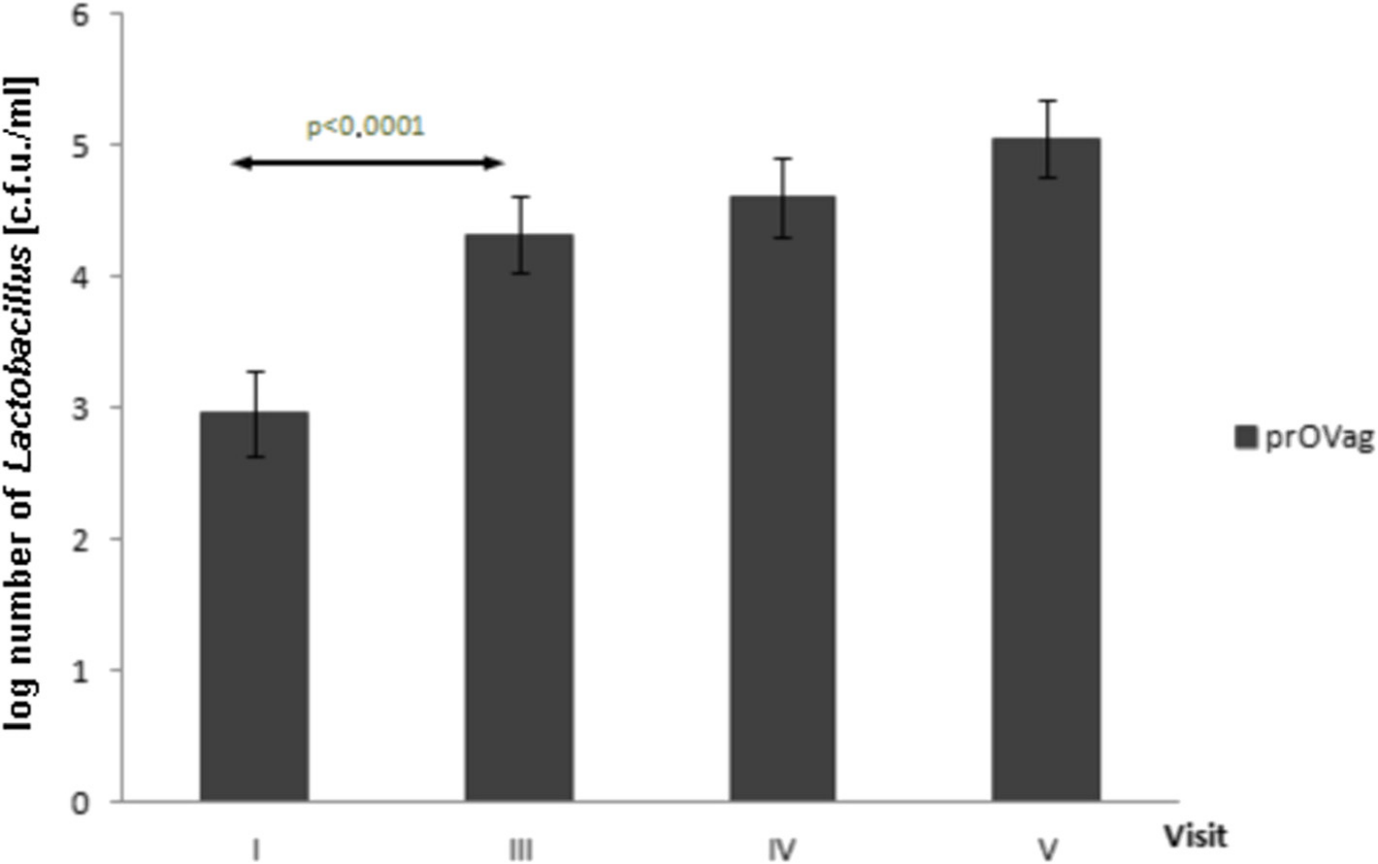
Effect of prOVag application on total numbers of vaginal lactobacilli during consecutive visits

**Fig. 7:**
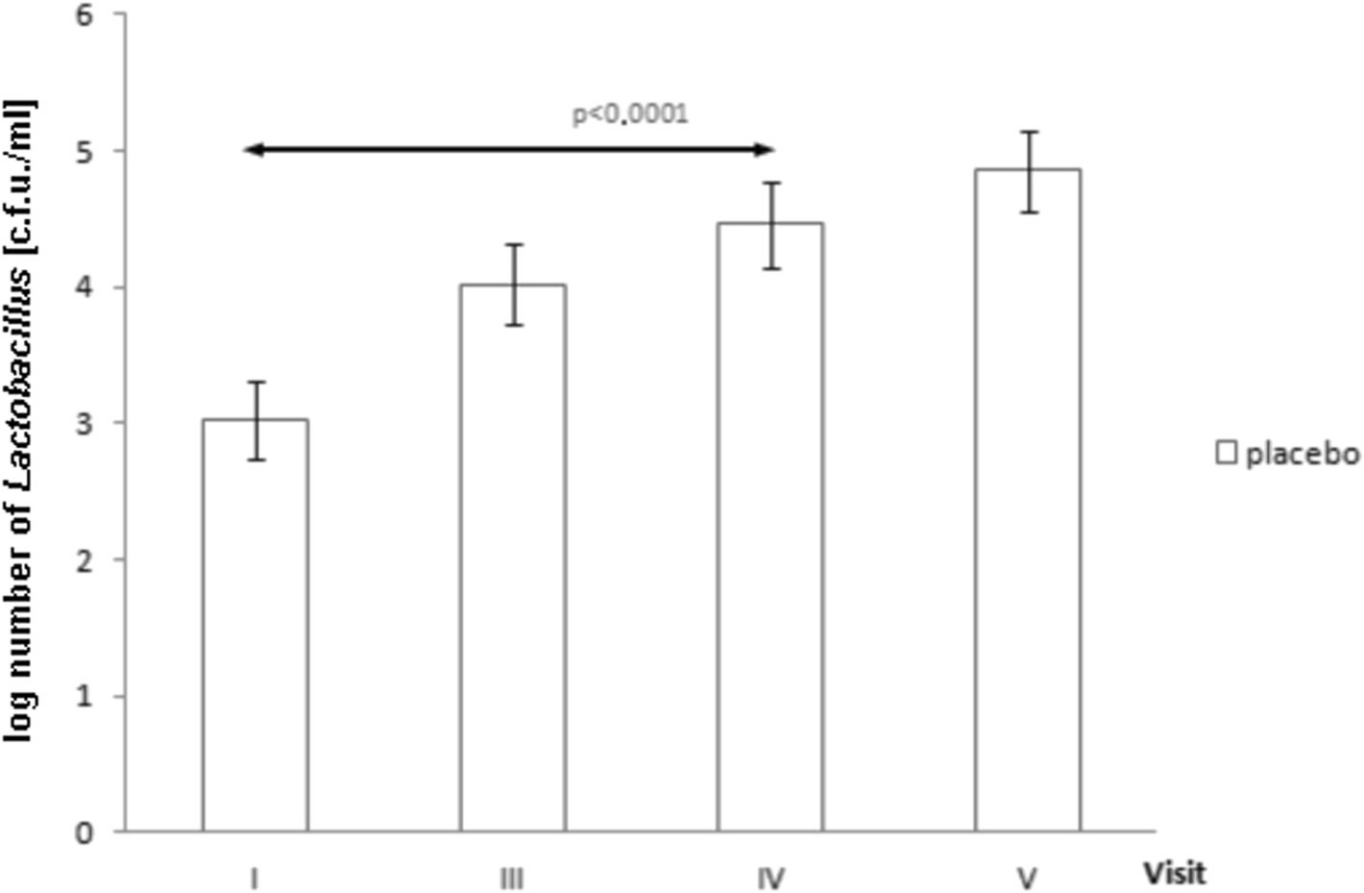
Effect of placebo application on total numbers of vaginal lactobacilli during consecutive visits

Total counts of vaginal *Lactobacillus* increased in both groups during the study period (*f*_placebo_ = 7.0664, *p*_placebo_ < 0.0001; *f*_provag_ = 8.9136, *p*_provag_ < 0.0001). *Post-hoc* Tukey’s tests revealed that a significant increase in *Lactobacillus* numbers *vs*. visit I was observed an average of one menstrual cycle earlier (visit III) in the prOVag (Fig. 6) than in the placebo group (visit IV; Fig. 7).

## Discussion

In the present study, we examined effects of the standard antibiotic treatment together with oral probiotic intake on recurrences of BV observed as clinical symptoms of vaginal infection and disturbances in the composition of vaginal microbiota. The number of subjects enrolled, methods used to evaluate vaginosis, and results obtained address the calls for well-designed, placebo-controlled clinical studies involving large patient groups to examine the use of probiotics to treat BV that have been put forward by the authors of reviews and meta-analyses [18].

Our study protocol focused on demonstrating that the use of the prOVag probiotic preparation simultaneously with standard treatment for BV reduced the rate of relapses, assessed using clinical and microbiological criteria, *versus* the standard treatment alone.

This study was conducted in several clinical centers located in southern Poland using the same protocol. In order to ensure the study consistency with the protocol and conform to Good Clinical Practices (ICH-GCP), the whole course of the trial was monitored by third party monitoring entities (CRO), which guaranteed protection of participants' rights, their safety, and completeness, reliability, and quality of the obtained data. Moreover the study was constantly under supervision of Medical and Microbiological Experts. Microbiological analyses were performed in one central laboratory possessing accreditation ISO certificate Nr AB 1428 for performing tests for vaginal infections. Obviously, more clinical studies done in different countries would be desirable to confirm and validate clinical efficacy of prOVag in women with various types of the vaginal microbiota alterations related to BV or AV [25].

Our trial involved a much larger number of participants than did previous studies. In addition, no previously published study used both widely accepted clinical (Amsel) and microbiological (Nugent score and pathogen count *vs. Lactobacillus* count) criteria. Our analysis demonstrated that the use of prOVag achieved a significantly longer interval between cure and the relapse of clinical symptoms of BV, compared with placebo. Because the criteria used for microbiological assessment are much stricter than those for clinical assessment, no such effect was observed for the interval between cure and microbiological relapse (i.e. redetection of abnormal vaginal microflora). Microbiological analysis of vaginal smears was used to demonstrate colonisation of the vaginal epithelium by pathogens, expressed as abnormal composition of vaginal microbiota. This definition of colonisation implies that it precedes the onset of BV symptoms observed by patients and physicians, and explains the difference observed in the times to microbiological and clinical relapse.

The presented study also included women with AV. To date, no other study has evaluated the effect of oral probiotics on the course of AV. We found that the use of prOVag significantly delayed the clinical relapse of AV symptoms by an average of 31 days (up to 76 %) in patients who used a targeted antibiotic, i.e. those in whom pathogens responsible for AV (*S. agalactiae*, *E. coli*, *E. faecalis*) were detected, compared with placebo.

The analysis of changes in vaginal pH and Nugent score values between the first and fifth visit confirmed the efficacy of prOVag compared with placebo. Women using prOVag showed continuous improvement in these parameters from the first to fifth visits. Changes in vaginal microflora were associated with increased *Lactobacillus* counts, which decreased the pH of the vaginal environment and inhibited the growth of pathogens. The population of *Lactobacillus* was restored significantly faster after standard treatment in women using prOVag than in the placebo group. Similar results were obtained in our previous open-label study of the use of prOVag by women with intermediate/abnormal vaginal flora and no clinical symptoms [26].

Thus, the use of prOVag contributes significantly to the maintenance and prolongation of treatment outcomes achieved with targeted antibiotics in patients with AV/BV, selected on the resistance of vaginal pathogens to chemotherapeutics. No report of the possible use of probiotic preparations to enhance and prolong the treatment outcome of AV achieved with different antibiotics has been published previously. The same effect, as a total novelty, was achieved in women treated with targeted oral clindamycin due to the resistance of *G.vaginalis* strains to routinely used metronidazole. Because the number of resistant *G.vaginalis* strains has increased recently [27], the possibility of using prOVag in such cases offers considerable benefits for a growing number of patients. Thus, the effect of prOVag is independent of the administered antibiotic and relies on efficient and persistent restoration of the normal composition of vaginal microflora, which naturally protects the vaginal epithelium from colonisation by pathogenic factors [28].

From clinical point of view, since metronidazole, the most commonly prescribed antibiotic to treat BV, causes only short-term suppression of the *G.vaginalis* populations in the vagina as revealed recently by Meyer et al. [29], the combined therapy with this antibiotic plus prOVag can successfully eliminate anaerobic bacterial pathogens responsible for BV, thereby delaying or preventing the relapse of vaginal inflammation. Their paper in fact augments importance of the combined treatment with metronidazole plus oral probiotic in long-term prevention of recurrent BV.

Although *Lactobacillus* strains used in prOVag are susceptible to clindamycin *in vitro*, their uninterrupted application throughout the study has sustained vaginal colonization. In fact, little is known about bactericidal effect of clindamycin against lactobacilli. Aroutcheva et al. found that the mean MIC values for studied lactobacilli was as high as 1000 ug/ml and stated that only high concentrations of clindamycin achieved in the vagina might be inhibitory for *Lactobacillus*. Clindamycin was rapidly eliminated from the vagina, within 3–8 days, after local administration [30]. Results shown by Eriksson et al. indicate that local treatment with probiotic lactobacilli could be problematic if carried out within 5 days after cessation of clindamycin treatment, but this is not in the case of the oral application [31]. Moreover, we have demonstrated before that maximal vaginal colonization with the prOVag strains was recorded between 31st and 60th day after the initiation of the treatment [26]. Most probably, an efficient colonization of the colon and rectum creates dense populations of the lactobacilli, which then constantly move to vagina. On the other hand, our lactobacilli were resistant to metronidazole and thus oral application of this drug should not influence their populations in rectum and vagina which is quite important from the practical point of view in the light of the recent findings reported by Mayer et al. [29].

Vaginal delivery of the probiotic strains via oral/anal route seems to be well documented in the light of the papers published by Reid’s group and us [26, 32]. It may be therefore proposed to consider probiotic supplementation of the antibiotic therapy for recurrent BV as a new adjuvant treatment for preventing recurrences.

## Conclusions

The analysis of efficacy parameters demonstrated that prOVag significantly lengthened the time to relapse of BV/AV clinical symptoms compared with placebo. In addition, the effect of prOVag was more powerful in patients using a targeted antibiotic (i.e.those diagnosed with AV and/or metronidazole-resistant *G.vaginalis*) than in the per-protocol population. In such cases, prOVag lengthened the time to BV relapse by as much as 76 %. We also found that the use of prOVag reduced and maintained vaginal pH and the Nugent score and stimulated an increase in the *Lactobacillus* count in vaginal flora during standard treatment, thereby helping to sustain the effect of BV/AV treatment. This study demonstrated the safety and efficacy of prOVag in preventing BV recurrence, as this product contributes to the improvement of clinical and microbiological parameters, thereby lengthening the remission period.

## Additional file

Additional file 1:
**Consort 2010 checklist of information to include when reporting a randomised trial [** 33 **]*.** (DOC 214 kb)
